# Testing the Geroscience Hypothesis

**DOI:** 10.1093/geroni/igaf122.403

**Published:** 2025-12-31

**Authors:** Sara Espinoza

## Abstract

Aging is the most significant risk factor for many chronic diseases and geriatric syndromes. The geroscience hypothesis posits that by targeting underlying cellular and molecular mechanisms of aging, it is possible to delay age-related diseases and thereby extend healthy years of life spent free of multimorbidity, disability, and frailty (healthspan). Towards this end, and informed by scientific discoveries in the biology of aging field, translational studies are ongoing to test the geroscience hypothesis by examining potential gerotherapeutics to extend healthspan. This session focuses on recent research to test the geroscience hypothesis, including studies of rapamycin and novel agents in preclinical models and human studies, including clinical trials.

